# Nitrogen Input Alters Root Exudation of *Kandelia obovata* and Nitrogen Cycling in Constructed Mangrove Wetlands

**DOI:** 10.3390/plants15121851

**Published:** 2026-06-15

**Authors:** Peiyin Wang, Dongpeng Yin, Guiping Fu, Xiaohan Yi, Zhipeng Guo

**Affiliations:** Shenzhen Key Laboratory of Marine Bioresource and Eco-Environmental Science, College of Life Sciences and Oceanography, Shenzhen University, Shenzhen 518055, China; wangpeiyin2022@163.com (P.W.); yindongpeng_1995@163.com (D.Y.); xiaohanyi1003@163.com (X.Y.); guozp@szu.edu.cn (Z.G.)

**Keywords:** mangrove wetland, root exudates, dissolved inorganic nitrogen, nitrogen cycle, microorganisms

## Abstract

The role of mangrove root exudates in mediating the nitrogen cycle, particularly under high dissolved inorganic nitrogen (DIN) input, in coastal ecosystems remains unclear. This research investigated variation in the root exudates, and nitrogen transformation and output, in constructed mangrove wetlands planted with *Kandelia obovata* under high, moderate, and low nitrogen-input levels (PCWs-H, PCWs-M, and PCWs-L, respectively). PCWs-H promoted increased root density and biomass accumulation, enhancing soil nitrogen sequestration, whereas PCWs-L induced greater specific root length, specific root surface area, and number of root tips. These changes directly influenced denitrification efficiency. Hydroxymethoxyphenylcarboxylic acid-*O*-sulfate and Arg-Ser released in root exudates under PCWs-H might act as potential denitrification inhibitors, thereby suppressing denitrifiers and impairing dissolved nitrogen purification. Elevated nitrogen loading predominantly limited denitrification, resulting in relative NO_3_^−^-N removal rates of PCWs-H < PCWs-M < PCWs-L (*p* < 0.05). Compared with PCWs-H and PCWs-L, the enhanced soil organic nitrogen storage under PCWs-M was associated with flavonoids in root exudates. Metagenomic analysis showed that denitrification was the dominant nitrogen removal pathway. Nitrogen loading influenced the effects of root exudates on the microbial community. Under PCWs-H, triterpenoids promoted *nor*BC and *nir*K/S abundance but depressed *amo*ABC abundance. Sterols and flavonoids in exudates under PCWs-L depressed *nos*Z abundance, instead activating dissimilatory nitrate reduction to ammonium. Compared with PCWs-H and PCWs-L, N_2_O emissions were minimal under PCWs-M. This study revealed that mangrove root exudates mediate the nitrogen cycle in mangrove wetlands, providing a theoretical basis for local authorities to manage DIN inputs and mitigate N_2_O emissions.

## 1. Introduction

Mangrove wetlands serve a vital function as coastal blue carbon reservoirs in the mitigation of global climate change [1,2,3]. The transport of dissolved inorganic nitrogen (DIN) from river catchments to coastal sediments on a global scale continuously influences nitrogen (N) cycling within mangrove wetlands and subsequently exacerbates nitrous oxide (N_2_O) emissions associated with the N cycle [3,4,5]. Mangrove wetlands in China are subject to increasing DIN pollution. MEE (2024) reported that the Pearl River Estuary is among the most severely eutrophic marine areas in China, and DIN was identified as the primary polluting factor, particularly in Shenzhen Bay [6]. Thus, the impact of DIN pollution on mangrove N cycling, and especially N_2_O emissions, presents challenges for the regulation of greenhouse gas emissions by mangrove wetlands in China.

Pollution by DIN adversely affects soil nutrient composition and plant growth. Increased soil DIN significantly enhances plant biomass in the short term, which improves a plant’s ability to absorb and utilize nutrients, thereby enhancing carbon sequestration in the ecosystem [7,8]. However, long-term N input can enable plants to obtain sufficient nutrients from the surface soil, leading to gradual root-system atrophy and wetland degradation and a decrease in carbon sequestration capacity [9]. These studies demonstrate the effects of sustained N enrichment on mangrove roots. Moreover, DIN pollution influences the N cycle in mangrove wetlands, which comprises three biogeochemical transformation processes: N input, transformation (i.e., N removal), and output [10,11,12]. Nitrogen transformation primarily occurs through various biochemical reactions, including microbial ammonification, nitrification, denitrification, dissimilatory nitrate reduction to ammonium (DNRA), and sulfate reduction [10,11]. In addition, physical processes, such as volatilization and adsorption, facilitate the transformation of different N compounds [12]. Nitrogen output, predominantly as N_2_O, is primarily associated with microbial denitrification. In other plant species (e.g., rice, sorghum, and *Brachiaria humidicola*), root exudates may cause significant alteration in the soil microbial community and thereby affect N transformation processes [13].

Root exudates are belowground allocation products of photosynthetically fixed carbon, serving as an organic carbon source for soil microorganisms and contributing to the vitality of the rhizosphere microecosystem [14]. Root exudates raise the total nitrogen (TN) concentration for N storage, of which total organic nitrogen (TON) is the primary component, thereby significantly influencing N cycling in wetlands [15]. Sucrose and glucose in root exudates from *Avicennia schaueriana* and *Rhizophora mangle* in Brazilian mangrove wetlands influence the abundance of bacterial genes encoding *nir*K (copper-containing nitrite reductase) and *nir*S (nitrate reductase). Thus, root exudates may act as an endogenous carbon source for heterotrophic denitrifiers, thereby regulating the distribution patterns of microorganisms in N-polluted waters [10]. The addition of organic acids and soluble sugars from root exudates of *Phragmites australis*, *Typha angustifolia*, and *Cyperus alternifolius* to constructed freshwater wetlands significantly enhanced the total N removal efficiency of the effluent by 47.1–58.67% compared with that of the control, whereas amino acid treatment resulted in a decrease in N removal [16]. Thus, different components of root exudates exert complex influences on rhizosphere N-cycling processes. In addition to modulating microbial distribution, root exudates act as biological nitrification inhibitors; for example, sorgoleone and sakuranetin from sorghum, brachialactone from forage grass, and 1,9-decanediol from rice inhibit hydroxylamine oxidoreductase and ammonia monooxygenase [17]. Root exudates may include biological denitrification inhibitors, such as procyanidin from *Fallopia* spp. [18]. Previous research on N cycling in mangrove wetlands primarily focused on microbial mechanisms, neglecting mangrove roots and their exudates [10,11,12], and largely examined terrestrial and freshwater plants [13,19]. Studies on the role of root exudates in increasing N_2_O emissions from mangroves in response to elevated DIN loads are notably lacking. Investigation of the impact of elevated DIN input on N cycling in mangrove wetlands, focusing on N removal, storage, and N_2_O modulation by root exudates, is crucial for understanding their important functions in biogeochemical cycles.

In this study, laboratory-scale constructed *Kandelia obovata* wetlands were established under three DIN inputs with the following aims: (1) evaluate the variation in root morphology and root exudates of *K. obovata* under different N inputs; (2) assess the impact of root exudates on denitrification efficiency and vertical N distribution in the wetland soil; and (3) elucidate the microbial mechanisms by which root exudates influence N cycling. The results provide a theoretical foundation for managing DIN input levels, improving N removal, increasing N storage, and decreasing N_2_O emissions mediated by microbial activity modulated by mangrove root exudates.

## 2. Materials and Methods

### 2.1. Design and Operation of Constructed Wetlands

**Figure 1:**
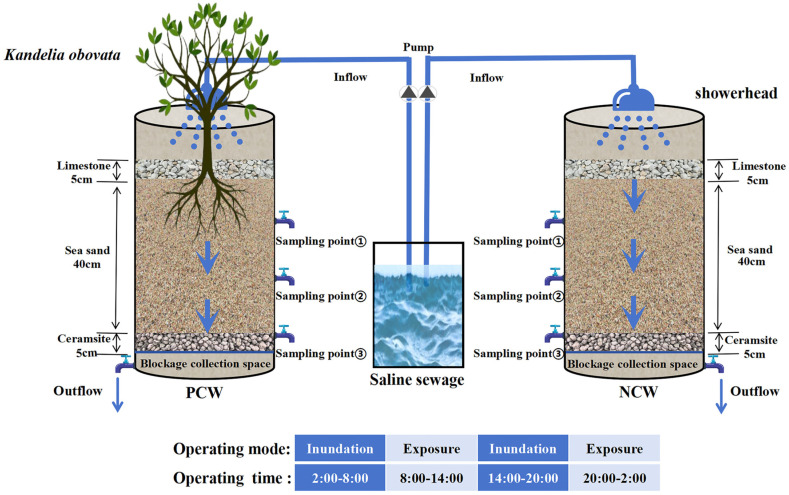
Design and operational schematic of PCWs and NCWs.

In April 2024, natural light laboratory-scale planted constructed wetlands (PCWs with *K. obovata*) and non-planted constructed wetlands (NCWs) were established (Figure 1). The wetlands were subjected to low (4.17 ± 1.01 mg/L NO_3_^−^-N), moderate (8.67 ± 0.99 mg/L NO_3_^−^-N), or high (17.72 ± 1.37 mg/L NO_3_^−^-N) N input, with three replicates per level, based on the N pollution conditions prevailing in western Shenzhen Bay coastal waters [20].

Each wetland chamber was constructed from polymethyl methacrylate (height 65 cm, inner diameter 20 cm) and filled with 5 cm of limestone (1 cm particle size), 40 cm of sea sand (1 mm particle size), and 5 cm of ceramsite (30–50 mm particle size). The sea sand and ceramsite were mixed with intertidal mangrove sediment collected from the Xichong Mangrove Wetland Park (volume basis: 3.3:1) as the inoculum source of microorganisms. A single *K. obovata* seedling (20–25 cm tall) was planted at approximately 10 cm depth in each PCW. Sampling ports 1, 2, 3 were set at 25 cm, 40 cm, 55 cm from the top of each wetland chamber, and an outflow port was used to drain water. A permeable baffle (2 μm pore size) was installed 5 cm above the chamber base to prevent clogging of the drainage port. An outlet (20 mm diameter) was connected to a drainpipe to control the internal water level. Simulated N-loaded seawater was injected via a peristaltic pump (LS PLUS-B193, Kamoer, Shanghai, China). The operation followed a semi-diurnal tidal-flow pattern, with water inflow occurring at low tide at 2:00 and 14:00 and water discharge occurring at high tide at 8:00 and 20:00 (Figure 1, Table 1). This operation was based on the natural tides along the western coast of Shenzhen Bay.

### 2.2. Water Quality Analysis

During operation of the constructed wetlands, inflow and outflow water quality after the first 6 h semi-tide was monitored every 2 days. Dissolved oxygen (DO) and pH were measured on-site with an Orion Star A meter (Thermo-Fisher Scientific Orion Star A121, Waltham, Massachusetts). After filtration, NH_4_^+^-N was analyzed using salicylic acid spectrophotometry (Chinese Standard HJ 536-2009), NO_2_^−^-N was determined by naphthylethylenediamine spectrophotometry (Chinese Standard GB 7493-87), and NO_3_^−^-N was analyzed by ultraviolet spectrophotometry (Chinese Standard HJ/T 346-2007). Total organic carbon (TOC) was determined using a Multi N/C 2100 TOC analyzer (Analytik Jena AG, Jena, Germany).

### 2.3. Detection of Nitrous Greenhouse Gas

**Figure 2:**
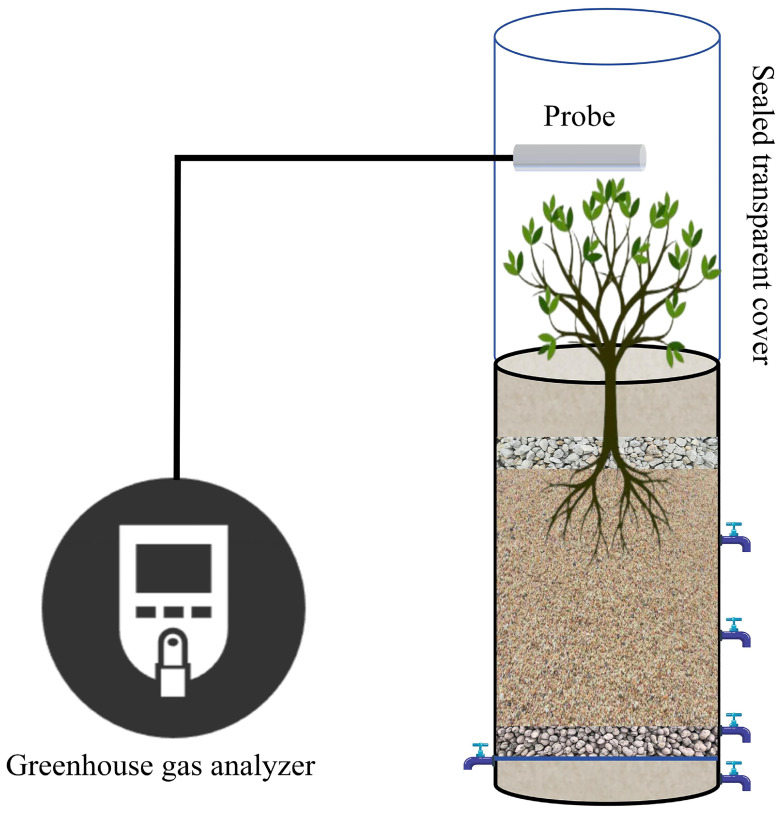
The monitoring system for N_2_O emissions from PCWs.

The 24 h N_2_O flux from the PCWs was measured using the closed static chamber method in the final operational week (64–70 Days). Gas samples were collected every other day on 64 Day, 66 Day, and 68 Day, yielding a total of three independent flux measurements. To prevent N_2_O from overflowing, the PCWs were maintained in a flooded state without water inflow, and the sampling and outflow ports were closed to avoid drainage. A cylindrical plexiglass cover (20 cm diameter, 65 cm height) created a sealed space over the wetland chamber (Figure 2). A small hole allowed probe insertion. The TD600-SH-B-M3 portable gas analyzer (Beijing Tiandi Shouhe Technology) continuously detected N_2_O for 24 h with data recorded at 30-min intervals. The air temperature during measurements ranged from 12.4 °C to 24.2 °C.

### 2.4. Soil Collection and Analysis

On the last day (70 day) of the PCWs and NCWs operations, samples of the surface (15 cm), middle (30 cm), and deep (45 cm) soils were collected for each N-input treatment. Impurities (e.g., stones and roots) were removed. Approximately 100 g soil was air-dried, ground, and sealed for storage to determine the soil DOC, NH_4_^+^-N, NO_2_^−^-N, NO_3_^−^-N, and total organic nitrogen (TON) using a CleverChem 380 automatic discrete analyzer (Dechentreiter GmbH, Hamburg, Germany).). The total inorganic nitrogen (TIN) comprised NH_4_^+^-N, NO_2_^−^-N, and NO_3_^−^-N, whereas TN comprised the sum of TON and TIN. The remaining soil samples were stored at −80 °C for microbial analysis.

### 2.5. Root Morphology and Exudate Analysis

Following the 70-day experimental period, *K. obovata* plants were carefully removed from the soil and placed into hydroponic culture for between 1 and 3 days. Root exudates from plants under each treatment (PCWs-L, PCWs-M, and PCWs-H) were collected using the hybrid method of Williams et al. [21]. The root exudation rate was determined by measuring the soluble organic carbon (DOC) release rate [*R*_DOC_; mg/(g·h)]. Exudates were extracted with ethyl acetate and analyzed with a Xevo G2-XS QTof mass spectrometer (Waters). The data were processed with Progenesis QI software (v3.0), significant differential metabolites were screened (*p* ≤ 0.05), and compounds were identified using the Human Metabolome Database (HMDB; http://www.hmdb.ca) and LIPID MAPS (https://lipidmaps.org/).

Following the method of Xu et al. [22], intact roots were rinsed, separated from the aboveground organs, and imaged with a MICROTEK scanner (Shandong Leander Intelligent Technology Co., Ltd., Weifang, China). Root traits were analyzed using IN-GX02 software (V7.041), with correction for crossed roots and exclusion of debris with an aspect ratio < 4. After analysis, the roots were blotted dry, weighed, dried at 60 °C for 48 h, and reweighed to determine the dry matter content. The specific root length (SRL; cm/g), specific root surface area (SRSA; cm^2^/g), and root tissue density (RTD; g/cm^3^) were calculated.

### 2.6. DNA Extraction, Sequencing, and Functional Gene Analysis

DNA was extracted from the soil samples and sequenced on an Illumina NovaSeq 6000 platform (Guangzhou Magigene Biotechnology Co., Ltd., Shenzhen, China). Raw reads were assembled de novo and genes were predicted using MEGAHIT (v1.2.9). Gene clustering and redundancy reduction were performed using Linclust as described. Non-redundant unigene sequences were aligned against the NCBI NR database using BLASTP. For functional annotation, the predicted protein sequences were searched against the KEGG, eggNOG, and CAZy databases (e-value 1 × 10^−5^). Nitrogen-cycle-related genes were screened based on the DiTing database. Gene abundances were analyzed using stacked bar plots and metabolic cycle profiling.

### 2.7. Statistical Analysis

Data from the PCWs and NCWs were processed using Excel 2021. Analysis of variance and correlation analyses were conducted using SPSS 25.0, with *p* < 0.05 and *p* < 0.01 indicating significance. GraphPad Prism 10 and Origin 2024 64Bit were used for visualization of the results.

## 3. Results and Discussion

### 3.1. Effects of N Input on Root Morphology and Exudation in Planted Constructed Wetlands

Nitrogen input significantly affected the root morphology of *K. obovata*. A greater total root length, SRL, SRSA, and root exudation rate were recorded in PCWs-L compared with PCWs-M and PCWs-H (Table 2). The high N-input level reduced the number of root tips (*p* < 0.05), whereas root density and biomass were increased in PCWs-H. These findings suggest that the N-input level influences nutrient allocation to *K. obovata* roots rather than merely suppressing growth. The increases in root length and surface area in PCWs-L enhanced nutrient acquisition, whereas the nutrient-rich PCWs-H supported greater biomass. These results are consistent with previous findings that N input (20 mg/L NH_4_^+^-N and 5 mg/L NO_3_^−^-N) boosts mangrove growth [12]. However, in terrestrial plants, such as rapeseed, low nitrate enhances the root-to-shoot ratio [9], and high N input causes root atrophy [23]. *Kandelia obovata* showed greater tolerance of nutrient pollution, efficiently assimilating N and phosphorus, which, in turn, facilitated sustained biomass accumulation.

The DOC concentration decreased with increase in N-input level, following the trend PCWs-L > PCWs-M > PCWs-H, although the differences between treatments were non-significant (*p* > 0.05). Thus, *K. obovata* increases root exudation under a low N concentration to enhance N acquisition by attracting beneficial microorganisms and improving nutrient solubilization and stress tolerance [24]. Conversely, high N availability likely suppresses exudation as *K. obovata* reduces investment in nutrient-mobilizing strategies.

A significant negative correlation between the number of root tips and DOC exudation (*r* = −0.562, *p* = 0.015) in *K. obovata* was observed. This result challenges the viewpoint that fine-root tips are the main sites of exudate release. Although an increase in root branching led to greater total root length and surface area, it did not correspond to higher carbon exudation. This suggests that the plant may prioritize growth and structural expansion over root exudation, indicating a shift in metabolic resource allocation [25]. These findings highlight that a greater abundance of root tips may not enhance root exudation activity but rather reflect a focus on root development [23].

### 3.2. Composition and Effects of Root Exudates

**Figure 3:**
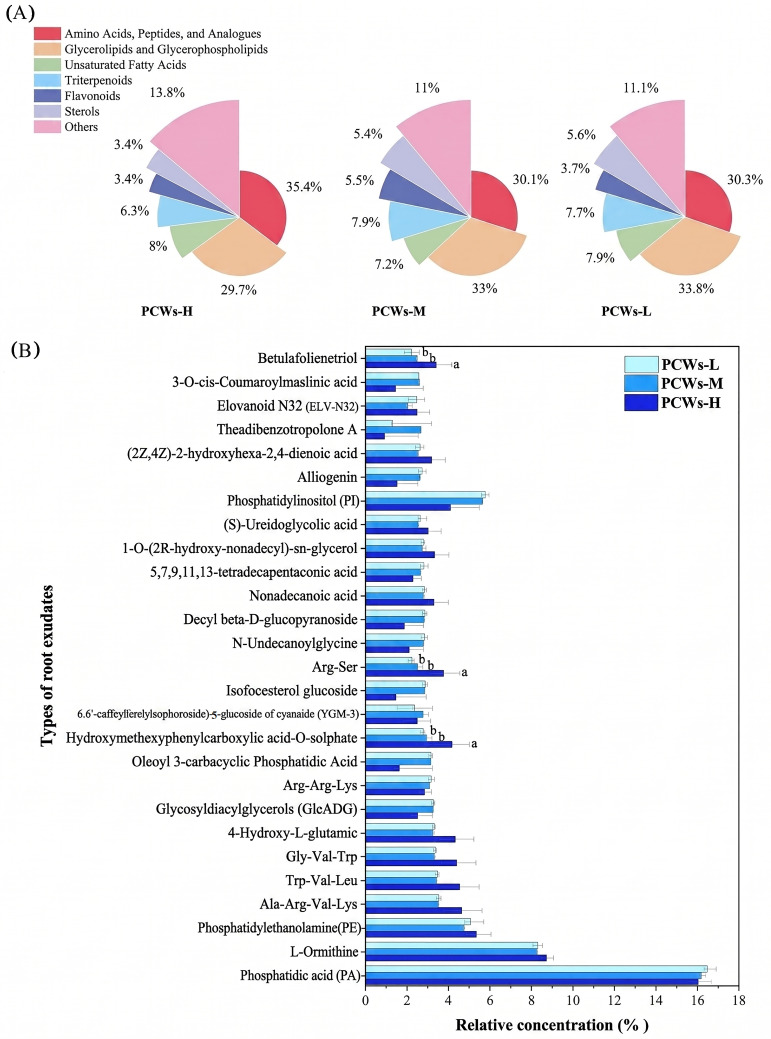
Root exudates and their relative abundance in PCWs and NCWs under different nitrogen input: (**A**) Major classes; (**B**) Subclasses. The different letters indicate significant differences (*p* < 0.05); the absence of letters indicates no significant differences.

Sixty-three metabolites were identified in the root exudates across all treatments, of which 27 showed differential abundance and high confidence scores. The metabolites were classified into six categories; those with relative abundances of less than 3% were labeled as “Other” (Figure 3A). An increase in N availability enhances plant N metabolism, leading to greater synthesis and exudation of N-rich compounds, including amino acids and peptides [24]. The highest amino acid and derivative content was detected in PCWs-H (35.4%; Figure 3A), consistent with previous findings on N-dependent amino acid exudation [26] and the suppression observed under a low N-input level in maize [19]. Notably, Arg-Ser concentration in PCWs-H significantly surpassed that of the other treatments (*p* < 0.05; Figure 3B). High N availability might induce phosphorus stress, prompting elevated exudation of Arg to alleviate phosphorus limitation by improving phosphorus acquisition, as reported for Chinese fir under low-phosphorus stress [27]. In addition, root-exuded peptides may be hydrolyzed by soil enzymes to release amino acids, which undergo ammonification to produce NH_4_^+^-N [24], which is subsequently nitrified to NO_2_^−^-N, increasing the soil TIN. Thus, root-derived amino acids and peptides are crucial N sources that drive microbial N metabolism.

Glycerolipids and glycerophospholipids exhibited the second-highest relative abundance (29.7–33.8%) (Figure 3A), consistent with previous findings that lipids and lipid-like molecules predominate in the rhizosphere soil of *Phragmites communis* and are associated with plant signaling and defense responses [28]. Among these compounds, phosphatidic acid (PA) was the most abundant compound detected (Figure 3B). As a crucial intermediate in lipid biosynthesis, PA plays important roles in plant responses to biotic and abiotic stresses [28]. For example, PA can modulate the root architecture in rice by suppressing lateral root formation while promoting the growth of finer seminal roots [27]. In the present study, PA might be associated with the regulation of root architecture.

Root-exuded flavonoid compounds in PCWs-M (5.5%) were increased in relative abundance compared with that in PCWs-H (3.4%) and PCWs-L (3.7%) (Figure 3A). Low N supply (0.5 mg/L NH_4_NO_3_) suppresses *Cyclocarya paliurus* plant growth and triggers reactive oxygen species (ROS) accumulation, thereby activating flavonoid synthesis. In contrast, high N input (15 mg/L NH_4_NO_3_) enhances N assimilation and redirects carbon allocation to growth instead of defensive root exudation [13]. This discrepancy with the present results, whereby moderate N input (8.67 ± 0.99 mg/L NO_3_^−^-N) induced the highest flavonoid exudation, likely stemmed from the unique adaptive strategies of mangroves. Flavonoid exudation in *K. obovata* associated with carbon and N metabolism plays a crucial role in regulating the osmotic balance and maintaining the water potential under salt stress [29]. The moderate N-input level in PCWs-M represented an optimal balance for acclimation to saline conditions. In contrast, under the low N-input treatment, carbon would be prioritized for maintenance metabolism, while under the high N-input level, suppressed ROS levels and promoted growth would reduce the demand for defensive root exudation.

The relative concentrations of hydroxymethoxyphenylcarboxylic acid-*O*-sulfate and betulafolienetriol were significantly higher in PCWs-H than in the other treatments (*p* < 0.05; Figure 3B). Hydroxymethoxyphenylcarboxylic acid-*O*-sulfate is an organic acid that might play roles in nutrient coordination, soil pH regulation, and plant metabolic homeostasis. Organic acids can exert allelopathic effects on microbial activity [30], including selectively inhibiting or stimulating certain microorganisms and altering the activity of enzymes involved in carbon, N, and phosphorus metabolism. Therefore, this compound may act as an allelopathic agent influencing microbial communities associated with carbon and N metabolism in PCWs-H.

### 3.3. Effect of Kandelia obovata Roots on Denitrification Efficiency

**Figure 4:**
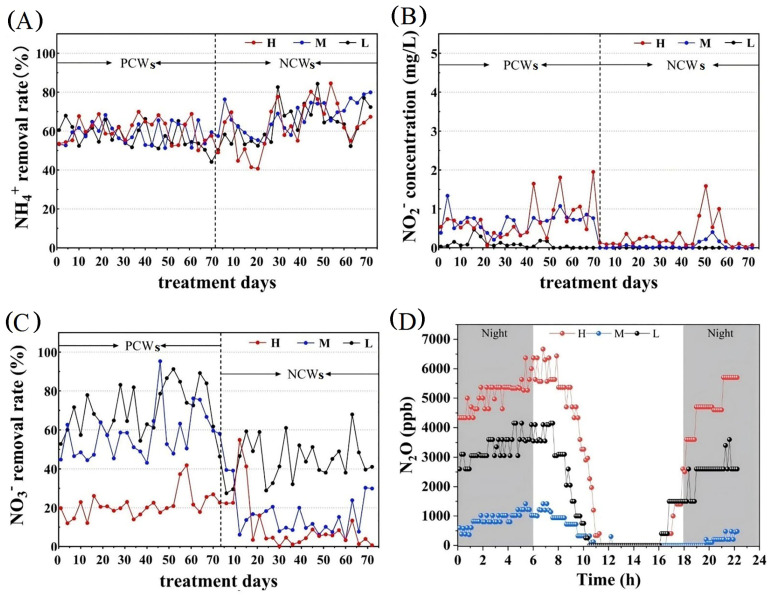
Effect of the *K. obovata* roots on denitrification efficiency during operation of PCWs and NCWs: (**A**) NH_4_^+^-N removal rate; (**B**) NO_2_^−^-N concentration; (**C**) NO_3_^−^-N removal rate; (**D**) N_2_O net flux in PCWs.

Comparison of the N forms in the PCWs and NCWs revealed a significant influence of root presence on wetland denitrification efficiency (Figure 4). The highest NH_4_^+^-N removal rate was observed in PCWs-H (60.06% ± 8.21%), followed by PCWs-M (58.68% ± 8.70%) and PCWs-L (57.96% ± 8.09%). In the NCWs, the ranking was NCWs-M (66.82% ± 7.27%) > NCWs-L (63.28% ± 7.54%) > NCWs-H (62.92% ± 9.08%) (Figure 4A). The presence of *K. obovata* roots in the PCWs significantly suppressed NH_4_^+^-N removal (*p* < 0.001), with no significant differences observed among the N-input treatments (*p* > 0.05). This lack of a concentration-dependent response likely stemmed from uniformly low NH_4_^+^-N loading (1 mg/L) across the treatments. The results suggest that nitrate-dominated N addition minimally affected nitrification activity in the mangrove wetland. Contrary to previous reports of higher NH_4_^+^-N removal in PCWs compared with unplanted controls (98.76% vs. 92.68%) [27], the present results indicated that DO concentrations were lower at root depth (30 cm) compared with those at 15 cm and in unplanted controls (Figure S1). Oxygen consumption from roots and microbial respiration exceeds radial oxygen loss, causing a net reduction of DO in the rhizosphere. Consequently, *K. obovata* inhibited NH_4_^+^-N removal (*p* < 0.001), with lower removal rates than reported values [31], reflecting limited nitrifier activity under low NH_4_^+^-N availability.

Accumulation of NO_2_^−^-N was detected in the wetland effluent under all N-input levels, with concentrations decreasing in the order PCWs-H (0.94 ± 0.56 mg/L) > PCWs-M (0.75 ± 0.44 mg/L) > PCWs-L (0.12 ± 0.07 mg/L) and NCWs-H (0.49 ± 0.37 mg/L) > NCWs-M (0.24 ± 0.19 mg/L) > NCWs-L (0.02 ± 0.04 mg/L). The PCWs and NCWs exhibited a consistent trend of an increase in NO_2_^−^-N accumulation with greater N loading (H > M > L), indicating that an elevated N concentration inhibits denitrification and leads to nitrite accumulation (Figure 4B). In the PCWs, NO_2_^−^-N accumulated primarily in the middle and lower soil layers (Figure S2), particularly in PCWs-M and PCWs-H, suggesting that denitrification was incomplete owing to low carbon:nitrogen (C/N) ratios and possible suppression of the oxygen-sensitive nitrite reductase (Nir) activity under the relatively high average DO concentration (3–3.5 mg/L). In contrast, NO_2_^−^-N accumulation in the unplanted controls was detected predominantly in the upper and middle substrate layers, likely resulting from partial nitrification–denitrification.

Over the 70-day operation, the NO_3_^−^-N removal rate decreased significantly with increase in influent N concentration (*p* < 0.05; Figure 4C). The removal rates in the PCWs and NCWs followed the order PCWs-L (68.03% ± 19.2%) > PCWs-M (56.50% ± 14.93%) > PCWs-H (22.54% ± 7.23%) and NCWs-L (44.48% ± 23.30%) > NCWs-M (16.17% ± 7.18%) > NCWs-H (5.12% ± 2.60%). PCWs-H showed lower NO_3_^−^-N removal than NCWs-L, indicating that the limiting effect of a low C/N ratio on denitrification exceeded the positive contribution of plant presence. The inferior NO_3_^−^-N removal in the NCWs might be attributed to the lack of available carbon, leading to greater reliance on nitrification rather than denitrification. These results underscored the critical influence of the C/N ratio on N removal efficiency in mangrove wetlands exposed to N-enriched seawater. The relatively high denitrification capacity observed in PCWs-L and PCWs-M may be mediated by aerobic denitrification, which is supported by the high DO environment (Figure S1).

The markedly higher NO_3_^−^-N removal rates in PCWs (22.54–68.03%) compared with NCWs (5.12–44.48%) highlights the role of root exudates in enhancing denitrification capacity. Correlation analysis identified 22 compounds in root exudates that were significantly associated with NO_3_^−^-N removal; sterol compounds exhibited a promotive effect, and triterpenoids showed an inhibitory influence (Figure S3). Certain compounds, such as sucrose, glucose, and oxalic acid, in root exudates may serve as effective carbon sources for *nir*K-type denitrifiers under a low C/N ratio, stimulating denitrifying bacterial activity and improving N removal [32]. Reduced NO_3_^−^-N removal in PCWs-H was correlated with an increase in amino acid exudation (Figure S3), consistent with the suppression of N removal in constructed wetlands following amino acid amendment [16]. In summary, root-derived organic carbon alleviated the C/N limitation in planted wetlands and mitigated the inhibition of denitrification under high N loading.

### 3.4. Effect of Kandelia obovata Roots on N_2_O Net Flux

The influence of *K. obovata* roots on N_2_O emission and its underlying mechanisms was examined by monitoring the N_2_O net flux over 24 h. The PCWs under each N-input level consistently exhibited elevated N_2_O emissions after sunset, which declined to near-zero levels by approximately 11:00 on average (Figure 4D). Several factors might explain this phenomenon. Because mangrove plants cannot directly utilize N_2_O, diurnal light variations play a critical role. At noon, peak sunlight stimulates vigorous photosynthesis, enhancing the oxygen-release capacity of the roots. This elevates DO concentrations in the wetlands, which stimulate aerobic denitrification and consequently reduces N_2_O emissions. Conversely, as light diminishes, root oxygen release decreases, leading to higher N_2_O emissions. This mechanism was supported by the DO profiles observed at a depth of 0–15 cm near the roots of *K. obovata* (Figure S1). The average N_2_O flux exhibited a non-linear relationship with N-input level in the order PCWs-H (3327.7 ± 2415.3 ppb) > PCWs-L (1754.6 ± 1580.7 ppb) > PCWs-M (413.0 ± 1134.7 ppb). This pattern is consistent with the 300% increase in N_2_O emissions from *K. obovata*-dominated mangroves under exogenous N enrichment [5]. The elevated emissions are likely driven by the provision of additional substrates for nitrification and denitrification in the sediments [1], as well as alleviated plant–microbe competition for N, resulting in enhanced microbial abundance and enzyme activity that promote N_2_O-producing metabolic pathways [33].

The N_2_O net flux in all treatments exceeded the 2023 global annual average atmospheric concentration (336.9 ± 0.1 ppb) [34], which can be attributed to the high N loading of the constructed wetlands. In contrast, nitrate scarcity in natural wetlands often limits denitrification completeness and N_2_O release [7]. In biological wastewater-treatment systems, N_2_O is predominantly derived from autotrophic nitrification and heterotrophic denitrification [35]. When hydroxylamine oxidation is incomplete or denitrification is truncated, the accumulation of intermediates can cause N_2_O to become both a product and a substrate in N transformation processes [36]. Thus, under high N availability, all systems exhibited substantial N_2_O fluxes, particularly in PCWs-H, which was indicative of incomplete N removal.

Denitrification is constrained primarily by carbon availability and DO. In the present study, root exudates from *K. obovata* served as a bioavailable carbon source that modulated the progression of denitrification. The higher root exudation rate in PCWs-L (Table 2) likely supplied sufficient carbon to support microbial N metabolism, facilitating denitrification and subsequent N_2_O conversion. Root-derived oxygen and organic compounds influence N_2_O dynamics via nitrification–denitrification coupling [37]. Eighteen compounds were significantly correlated with N_2_O flux (11 positively, 7 negatively; Figure S4), implying they potentially act as ecological regulators of denitrification. Differences in DO concentration among the treatments—in the order PCWs-M (3.13 ± 0.25 mg/L) > PCWs-H (3.08 ± 0.13 mg/L) > PCWs-L (2.76 ± 0.42 mg/L)—may also explain the reduced N_2_O emissions in PCWs-M, possibly by promoting aerobic denitrification under moderate oxygen availability.

In summary, a high N-input level promotes incomplete denitrification through reduction of the C/N ratio, root-exudate-mediated microbial regulation, and modulated radial oxygen loss, which collectively enhance N_2_O release from the wetland.

### 3.5. Effect of Kandelia obovata Root Exudates on Soil N Storage

#### 3.5.1. Storage of N Forms in the Soil

The total N pool in mangrove wetland soils comprises TON and TIN. Organic N can bind with soil aggregates, enhancing nutrient supply and supporting microbial growth and metabolism [38]. In contrast, inorganic N represents a more labile form that is readily transformed through microbial processes [39]. Consequently, organic N shows greater stability than its inorganic counterpart. However, organic N can be mineralized to inorganic N by microbial metabolism, and this decomposition process might release greenhouse gases (e.g., nitrogen oxides) [39], which has potential implications for climate change. The higher N_2_O release from PCWs-H may reflect the latter possibility. Thus, even at low concentrations, inorganic N plays a critical role in the wetland N cycle.

The average TN was significantly higher in PCWs (694.13 ± 179.95 mg/kg) than in NCWs (430.77 ± 170.76 mg/kg; *p* = 0.009; Table 3), indicating that plant presence facilitated N retention (*p* < 0.05). The TN among the PCWs decreased in the order PCWs-M > PCWs-H > PCWs-L. Soil N was predominantly present as TON, accounting for 96.3–99.7% of TN, which is higher than that reported by Chen et al. [40], indicating that the present wetland system favored organic N storage. The variation in TON corresponded closely with that of TN, and significantly higher TON was detected in PCWs-L than in NCWs (*p* < 0.05). In contrast, TIN constituted only 0.3–3.7% of TN, increased with elevation in N-input level, and was consistently higher in PCWs than in NCWs.

These results demonstrated that N input primarily influences soil TON dynamics, particularly in PCWs. The soil TON under a high N-input level was slightly lower than that under the moderate concentration, whereas TIN showed the opposite trend. This result may reflect that available TON in the soil was metabolized and decomposed by microorganisms into TIN. Notably, PCWs-L had the lowest soil TIN across all treatment groups and was lower than NCWs under the corresponding N-input level, likely because of the higher N removal efficiency under PCWs-L (Figure 4). Although elevation in N loading increased the absolute content of labile soil TIN, its low proportional contribution to soil TN indicates it has minimal short-term impact on the stability of the mangrove soil N pool.

The soil NH_4_^+^-N in PCWs increased gradually with elevation in N-input level (*p* < 0.05; Table 3), with the highest content recorded in PCWs-H (9.71 ± 1.16 mg/kg), followed by PCWs-M (7.92 ± 3.68 mg/kg) and PCWs-L (4.34 ± 1.72 mg/kg). In contrast, soil NH_4_^+^-N in NCWs was consistently low (0.57–0.67 mg/kg) and showed no significant response to N addition (*p* > 0.05). The soil NO_2_^−^-N was low in all treatments, remaining below 0.5 mg/kg, with no significant differences detected among N-input levels (*p* > 0.05). Although PCWs exhibited higher soil NO_2_^−^-N (0.025–0.399 mg/kg) than NCWs (0.009–0.017 mg/kg), suggesting a potential influence of plant presence and N-input level, the effect was non-significant (*p* > 0.05). Increase in N-input level significantly enhanced the soil NO_3_^−^-N (*p* < 0.05). Among the planted wetlands, soil NO_3_^−^-N decreased in the order PCWs-H (8.17 ± 0.47 mg/kg) > PCWs-M (5.51 ± 0.58 mg/kg) > PCWs-L (0.81 ± 0.34 mg/kg). Similarly, the soil NO_3_^−^-N in NCWs followed the order NCWs-H (7.80 ± 0.91 mg/kg) > NCWs-M (5.25 ± 1.09 mg/kg) > NCWs-L (4.30 ± 0.21 mg/kg).

The NH_4_^+^-N and NO_3_^−^-N removal rates in the water were significantly negatively correlated with their contents in the soil (*r* = −0.782, *p* < 0.001; *r* = −0.548, *p* = 0.006, respectively) (Table 3). Thus, denitrification by mangrove wetland microorganisms may play an important role in soil nutrient cycling. Contrary to a report of higher concentrations of NH_4_^+^-N than NO_3_^−^-N in mangrove soils affected by shrimp aquaculture effluent [8], the present study observed comparable contents of NH_4_^+^-N and NO_3_^−^-N in PCWs. This discrepancy might be explained by the dominant input of NO_3_^−^-N in the present experimental system, whereas mariculture discharges typically contain NH_4_^+^-N as the major inorganic N form. Therefore, the form of N loading in the influent was a critical factor influencing the composition of inorganic N in the soil.

#### 3.5.2. Vertical Distribution of N Forms in the Soil

**Figure 5:**
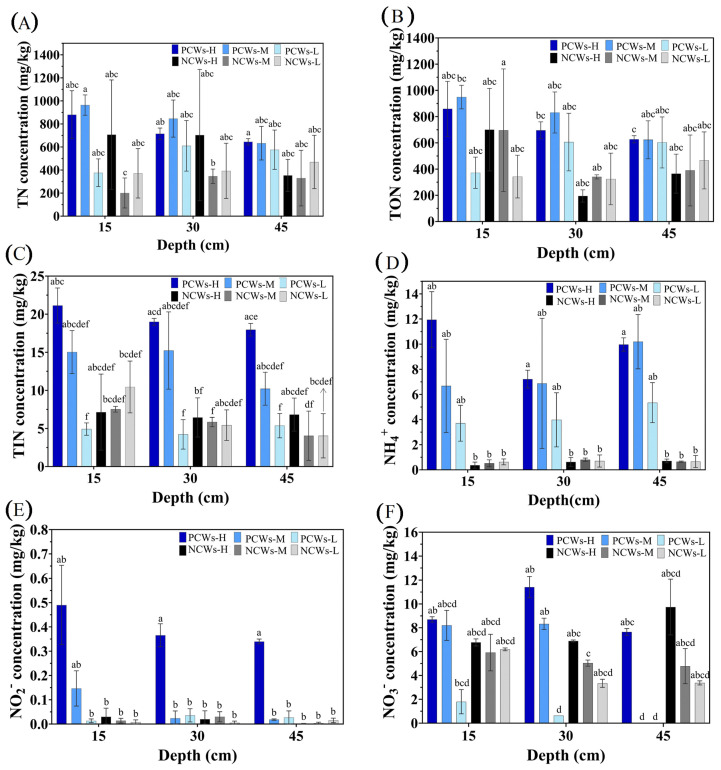
The changes in nitrogen storage at different soil depths in PCWs and NCWs under different nitrogen input levels: (**A**) TN; (**B**) TON; (**C**) TIN; (**D**) NH_4_^+^-N; (**E**) NO_2_^−^-N; (**F**) NO_3_^−^-N. The different letters indicate significant differences (*p* < 0.05).

The content of certain N forms in the soil did not change significantly with increase in soil depth (*p* > 0.05). However, the vertical distribution of soil TN differed among the treatments (Figure 5A). The soil TN in PCWs-H and PCWs-M decreased with increase in soil depth, whereas TN in PCWs-L increased in the middle and lower substrate layers, reflecting the release of organic N from the root zone at approximately 30 cm depth (Figure 5B). A similar trend was observed for soil TON. The lower C/N ratios in PCWs-H and PCWs-M likely promoted microbial mineralization of organic N. The soil TIN generally decreased with depth in all treatment groups (Figure 5C), with higher contents observed in PCWs-H and PCWs-M, consistent with the elevated influent N concentrations in these two treatments. The soil NH_4_^+^-N in PCWs decreased in the upper substrate layers and increased in the deeper layers (Figure 5D), reflecting nitrification in the surface soil and nitrate reduction in the subsurface layers. The NCWs maintained low NH_4_^+^-N contents at all soil depths. The soil NO_2_^−^-N was significantly higher in PCWs-H (*p* < 0.05; Figure 5E), consistent with NO_2_^−^-N accumulation in the water (Figure 4B) and the elevated N_2_O emissions (Figure 4D). This finding indicates that a high N-input level promotes NO_2_^−^-N accumulation, which might impair the ecological health of the wetland. The PCWs-H exhibited high soil NO_3_^−^-N contents at all soil depths (Figure 5F), resulting from the high N input and low N removal efficiency (Figure 4C). In contrast, soil NO_3_^−^-N was almost undetectable at 45 cm depth in PCWs-M and PCWs-L, consistent with the higher nitrate removal capacity in these treatments. In summary, the efficiency of N removal in the wetland is an important factor influencing the content and distribution patterns of soil inorganic N.

#### 3.5.3. Changes in Soil N Components Driven by Root Exudates

The variation in soil N components among the treatments was influenced by N-input level and was associated with root-exuded nitrogenous compounds and allelochemicals that recruit microbes that facilitate organic N storage. The PCWs-M exuded the highest relative amount of flavonoids (Figure 3A), and the soil TN and TON were also higher (Figure 5A,B), indicating that an excessively high or low N-input concentration reduces flavonoid secretion by *K. obovata*, thereby affecting soil N retention. This result is consistent with the effects of N supply on legume root exudates [41]. A Pearson correlation analysis indicated that root exudates had a more pronounced effect on soil NO_2_^−^-N and NO_3_^−^-N (Figure S4). Eleven compounds significantly increased, whereas 10 compounds decreased, the soil NO_2_^−^-N.

Hydroxymethoxyphenylcarboxylic acid-*O*-sulfate, Arg-Ser, and betulafolienetriol significantly promoted soil NO_2_^−^-N and NO_3_^−^-N (*p* < 0.05), effectively increasing TIN in PCWs-H, thus affecting the stability of the soil N pool in PCWs under a high N-input level. Root exudates can enhance TIN in low-fertility soil from less than 5 mg/kg to 12–18 mg/kg through primed mineralization and N fixation, indicating that plants can optimize TIN storage by modulating exudate composition (e.g., C/N ratio and organic acids) [42]. Compared with organic N, inorganic N is a more readily utilized N source for microorganisms, indicating that root exudates primarily act on the more labile components of the wetland N pool [13,33]. The impact of root exudates on the soil physicochemical properties regulates substrate availability for microbial metabolism, thereby influencing N transformation pathways [13].

The foregoing results indicate that even in a small constructed mangrove wetland, *K. obovata* significantly altered the soil environment and enhanced N storage. The highest TN was observed in PCWs-M, and the highest TIN was detected in PCWs-H. Increase in the N-input level significantly boosted soil TIN, NO_2_^−^-N, and NO_3_^−^-N (*p* < 0.01), which were associated with the high NO_3_^−^-N load in the influent. Consequently, long-term high N input might lead to accumulation of the harmful nutrient NO_2_^−^-N in mangrove wetlands, thereby adversely affecting mangrove growth and metabolism and potentially disrupting the ecological balance of mangrove and coastal ecosystems.

### 3.6. Analysis of Soil Microbial N Metabolic Potential

#### 3.6.1. Soil Microbial High-Throughput Sequencing and Community Structure Analysis

The number of operational taxonomic units and alpha-diversity index values (*S*_obs_, Shannon, and Chao1) were significantly higher in NCWs than in PCWs (*p* < 0.01; Table 4). Among the N-input treatments, only PCWs-H showed significantly lower values than PCWs-L (*p* < 0.01), and no significant differences were observed among the other treatment groups. Thus, high N input exerted a stronger inhibitory effect on microbial abundance in the presence of plants. The reduced microbial diversity in PCWs, which concurrently exhibited higher pollutant removal efficiency than NCWs, might reflect allelopathic root exudates that selectively enrich microbial taxa beneficial for N removal.

#### 3.6.2. Nitrogen Metabolic Functional Genes and N-Cycle Analysis

**Figure 6:**
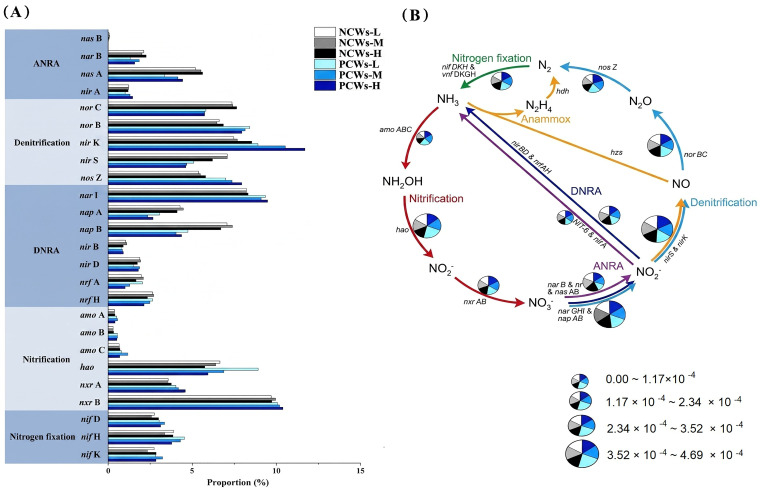
Effect of PCWs and NCWs under different nitrogen input levels: (**A**) the average abundance of microbial nitrogen metabolic functional genes; (**B**) the nitrogen cycle.

Functional genes associated with denitrification were significantly more abundant than those involved in nitrification (Figure 6A). Genes involved in hydroxylamine oxidation, nitrate reduction, nitrite reduction, and N_2_O reduction accounted for a relatively high proportion, indicating that these processes dominate the wetland N cycle (Figure 6B). Under aerobic conditions, ammonia-oxidizing microorganisms exhibited pronounced activity, resulting in notable proportions of *hao* (hydroxylamine oxidoreductase) and *nxr*B (nitrite oxidoreductase). The abundance of *hao* was significantly higher in PCWs-L than in the other treatments groups (*p* < 0.05), which reflected increased reliance on hydroxylamine metabolism by ammonia-oxidizing bacteria under a low N concentration. However, the low abundance of *amo*ABC (ammonia monooxygenase) and limited ammonium availability (1 mg/L NH_4_^+^-N) likely constrained the overall nitrification efficiency, which did not differ significantly among treatment groups (Figure 4A).

The reduction of nitrate to nitrite was prominent owing to high nitrate loading. Nitrite reduction, a critical intermediate step, was dominated by *nir*K, which showed significantly higher abundance in PCWs-H than in PCWs-L and NCWs (*p* < 0.05), correlating with the elevated NO_2_^−^-N substrate under the high N input. The widespread abundance of *nor*BC (nitrous oxide reductase) in all treatments contributed to the increase in N_2_O production (Figure 4D), consistent with the observation that N input elevates *hao* and *nir*K abundances but might not affect the abundance of other denitrification genes [12]. These findings suggest that the N form and concentration selectively influence microbial gene profiles.

In the absence of plant roots (NCWs), N-input level had minimal impact on functional gene abundance, consistent with previous findings that plant roots significantly affect microbial denitrification [43] and confirming the fundamental influence of plant roots on microbial function. *Kandelia obovata* root exudates included compounds that were promotive or inhibitory toward denitrification (Figure S5). Triterpenoids enhanced the abundance of *nor*BC and *nir*K/S but depressed the abundance of *amo*ABC and DNRA genes [i.e., *nir*BD and *nrf*AH (nitrite reductase)]. In contrast, sterols, flavonoids, and unsaturated fatty acids depressed the abundance of *nor*BC, *nir*K/S, and *nos*Z, likely because of allelopathy or substrate competition, while promoting DNRA and nitrification genes (*hao*). These interactions exhibited N-dependent regulation: high N input favored triterpenoid-mediated denitrification, promoting the abundance of *nir*K/*nor*BC, whereas low N input enhanced steroid-influenced nitrification and DNRA genes. The modulation of microbial N cycling by root exudates of *K. obovata* under inorganic N input provides a theoretical basis for the restoration of mangrove wetlands.

#### 3.6.3. Phylum- and Genus-Level Microbial Community Composition in N Metabolic Pathways

##### Phylum Level

**Figure 7:**
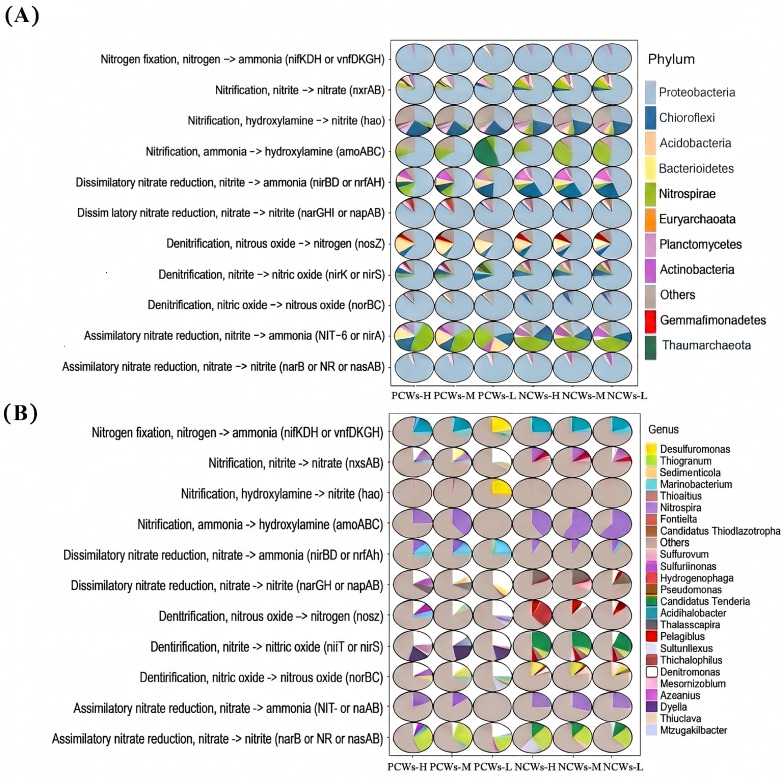
Comparison of nitrogen metabolic pathways and main microbial contributions in PCWs and NCWs: (**A**) Phylum level; (**B**) Genus level.

The microbial community composition at the phylum level reflected the effects of N input and plant presence on N metabolism. Proteobacteria was the dominant phylum (74.36–76.76%) across all treatment groups (Figure 7A), confirming its role in carbon and N transformation under high N concentrations [44,45]. Sterols, flavonoids, and unsaturated fatty acids enhanced the abundance of Chloroflexi, Bacteroidota, Acidobacteria, and Actinobacteria, whereas triterpenoids suppressed these phyla (Figures S6 and S7). Bacteroidota and Actinobacteria were more abundant in PCWs-L than PCWs-H, indicating the greater efficiency of nitrate removal in planted wetlands. Thaumarchaeota in PCWs-L, which carry *amo*ABC and *nir*K/S, contributed to ammonia oxidation and nitrite reduction, demonstrating the dual nitrification–denitrification capability under fluctuating redox conditions [46,47].

##### Genus Level

Genera responsible for N cycling and its regulation were influenced by root exudates. The microbial community composition in PCWs differed significantly from that of NCWs (Figure 7B). *Denitromonas* was the most abundant genus, contributing 17.6% to the DNRA pathway in PCWs-L, indicating the importance of aerobic denitrification [48]. *Denitromonas* abundance was positively modulated by ornithine and 3-*O*-*cis*-coumaroylmaslinic acid (Figures S8 and S9). *Marinobacterium* (abundance 3.6%), which played a critical role in DNRA in PCWs-L, is positively correlated with effluent NH_4_^+^-N concentration [45]. Negative correlations with hydroxymethoxyphenylcarboxylic acid-*O*-sulfate and Arg-Ser suggested that denitrification was inhibited in PCWs-H. *Desulfuromonas* was 1.7–4.8 times more abundant in PCWs-L and was positively correlated with NO_3_^−^-N removal (*r* = 0.850, *p* = 0.004), indicating the involvement of this genus in sulfur autotrophic denitrification [49,50]. The differences in composition of the denitrifying communities explained the higher nitrate removal rate in PCWs (Figure 4C). PCWs were dominated by *Denitromonas* (14.1–30.1%) and *Dyella* (6.2–7.8%), whereas NCWs relied on *Candidatus tenderia* (7.7–10.6%). *Dyella* abundance was positively correlated with theadibenzotropolone A and 3-*O*-*cis*-coumaroylmaslinic acid, which is consistent with findings that L-theanine reduces *Dyella* involvement in denitrification [51]. *Candidatus tenderia*, which is associated with nitrite reduction and assimilatory nitrate reductase activity in NCWs, was positively correlated with nitrate removal rate (*r* = 0.725, *p* = 0.027), indicating its role in N loss pathways.

## 4. Conclusions

Within the context of global warming and coastal eutrophication, this study dissected the complex interaction mechanisms among DIN input, *K. obovata* root exudates, and the microbial N metabolic functional genes participating in the N-cycling pathways, especially N transformation and output, in constructed mangrove wetlands. The high DIN input level promoted increases in root density and biomass accumulation, and root exudates enhanced the soil N sequestration. Potential denitrification inhibitors among the exudates, such as hydroxymethoxyphenylcarboxylic acid-*O*-sulfate and Arg-Ser, reduced the abundance of denitrifying microorganisms, whereas promotive compounds, such as triterpenoids, enhanced *nor*BC and *nir*K/S abundance but depressed *amo*ABC abundance; thus, these compounds modulated the abundance of denitrifying microorganisms and limited denitrification. Ultimately, the DIN removal rates decreased, resulting in a substantial increase in N_2_O emissions. Under low DIN input, *K. obovata* exhibited greater SRL, SRSA, and number of root tips, and sterols and flavonoids among the exudates inhibited *nos*Z abundance, which enhanced the efficiency of denitrification and the DIN removal rates and ultimately maintained a moderate level of N_2_O emissions. Under a moderate DIN input level, enhanced soil organic N storage was associated with flavonoids in the root exudates, and the root exudates maintained a moderate abundance of denitrification genes (*nir*K/S and *nar*I/*nap*AB), resulting in an intermediate level of microbial denitrification efficiency and minimal N_2_O emissions. The findings revealed that root exudation plays a fundamental modulatory role in the N-cycling processes of constructed mangrove wetlands through allelopathy, exudation of inhibitor compounds, and provision of carbon and N sources. Given that an intermediate denitrification efficiency and the lowest N_2_O emissions were observed under a moderate N input, we recommend that mangrove management authorities implement measures that prevent excessive N pollution while avoiding the establishment of overly strict regulatory standards.

## Figures and Tables

**Table 1:** Physicochemical indicators of influent and operational parameters for PCWs and NCWs.

Measured Indicators	Low Nitrogen	Medium Nitrogen	High Nitrogen
Salinity (‰)	10.0	10.0	10.0
Total organic carbon (TOC, mg/L)	26.63 ± 5.52	23.60 ± 7.62	24.92 ± 6.97
NH_4_^+^-N (mg/L)	1.02 ± 0.31	1.02 ± 0.35	1.1 ± 0.31
NO_3_^−^-N (mg/L)	4.17 ± 1.01	8.67 ± 0.99	17.72 ± 1.37
Hydraulic retention time (HRT, h)	12	12	12
Surface hydraulic loading rate (m^3^/m^2^∙d)	0.26	0.26	0.26
Organic loading rate (g TOC/m^2^∙d)	6.79	6.01	6.35
Nitrogen loading rate (g N/m^2^∙d)	1.32	2.47	4.79
C/N	5.13	2.43	1.32

**Table 2:** Root morphology and exudation rate of PCWs under different nitrogen input levels.

Root Morphology and Exudation Rate	PCWs-L	PCWs-M	PCWs-H
Total root length (cm)	476.66 ± 106.62 ^a^	261.05 ± 20.66 ^b^	267.10 ± 20.22 ^bc^
Surface area (cm^2^)	184.94 ± 21.64	139.35 ± 53.65	194.23 ± 62.02
Volume (cm^3^)	11.42 ± 5.76	10.10 ± 5.83	15.14 ± 6.47
Diameter (mm)	3.63 ± 0.79	2.83 ± 0.51	3.54 ± 0.51
Specific root surface area (SRSA) (cm^2^/g)	36.13 ± 3.75	28.27 ± 7.12	24.77 ± 5.98
Specific root length (SRL) (cm/g)	93.23 ± 19.63	69.80 ± 14.93	45.90 ± 8.98
Root density (g/cm)	1.15 ± 0.32	3.77 ± 1.79	2.64 ± 1.28
Biomass (g)	5.10 ± 0.26	4.37 ± 0.71	6.90 ± 1.14
Root tip number	582.83 ± 106.48 ^a^	370.67 ± 34.62 ^ab^	302.00 ± 53.00 ^b^
Dissolved organic carbon content in root exudates (DOC) (mg/L)	8.18 ± 2.26	5.24 ± 0.38	6.64 ± 1.30
Root exudation rate (*R*_DOC_) (mg C/(g·h)	8.74 ± 2.53	6.19 ± 0.83	4.24 ± 0.83

Note: The different letters indicate significant differences (*p* < 0.05); the absence of letters indicates no significant differences.

**Table 3:** Changes in nitrogen storage in PCWs and NCWs under different nitrogen input levels.

Wetlands	TN (mg/kg)	TON (mg/kg)	TIN (mg/kg)	NH_4_^+^-N (mg/kg)	NO_2_^−^-N (mg/kg)	NO_3_^−^-N (mg/kg)
PCWs-H	746.74 ± 95.35 ^ab^	727.38 ± 100.45 ^ab^	19.36 ± 1.19 ^a^	9.71 ± 1.16 ^a^	0.40 ± 0.07 ^a^	9.25 ± 0.47 ^a^
PCWs-M	814.68 ± 131.72 ^a^	801.18 ± 130.43 ^a^	13.49 ± 3.35 ^b^	7.92 ± 3.68 ^a^	0.06 ± 0.04 ^b^	5.51 ± 0.58 ^bc^
PCWs-L	520.99 ± 170.04 ^c^	526.84 ± 177.69 ^bc^	4.84 ± 1.45 ^d^	4.35 ± 1.72 ^b^	0.03 ± 0.02 ^bc^	0.81 ± 0.34 ^d^
Average value of PCWs	694.13 ± 179.95	685.13 ± 173.17	12.57 ± 6.54	7.33 ± 2.86	0.16 ± 0.19	5.19 ± 4.50
NCWs-H	588.02 ± 394.63 ^bc^	419.93 ± 170.11 ^bc^	6.80 ± 3.24 ^c^	0.57 ± 0.25 ^c^	0.02 ± 0.02 ^c^	7.80 ± 0.91 ^ab^
NCWs-M	298.48 ± 144.47 ^d^	475.28 ± 251.12 ^d^	5.81 ± 1.41 ^cd^	0.66 ± 0.15 ^c^	0.02 ± 0.01 ^c^	5.25 ± 1.09 ^bc^
NCWs-L	411.80 ± 228.61 ^cd^	377.83 ± 192.40 ^cd^	6.64 ± 2.77 ^d^	0.67 ± 0.40 ^c^	0.01 ± 0.01 ^c^	4.30 ± 0.21 ^c^
Average value of NCWs	430.77 ± 170.76	424.35 ± 170.55	6.42 ± 1.96	0.63 ± 0.12	0.01 ± 0.01 ^c^	5.78 ± 1.98

Note: The different letters indicate significant differences (*p* < 0.05); the absence of letters indicates no significant differences.

**Table 4:** High-throughput sequencing results and alpha diversity analysis of PCWs and NCWs under different nitrogen input levels.

Wetlands	Effective Sequences	OTUs	Alpha Indices				
			Sobs	Shannon	Simpson	Chao 1	Coverage
PCWs-H	40,834	3187 ^d^	3231.00 ^d^	6.10 ^d^	0.013 ^a^	4423.13 ^e^	0.97 ^a^
PCWs-M	40,834	3572 ^cd^	3574.15 ^cd^	6.39 ^cd^	0.009 ^ab^	4844.29 ^de^	0.97 ^a^
PCWs-L	40,834	3885 ^bc^	3886.40 ^bc^	6.53^c^	0.007 ^bc^	5305.48 ^cd^	0.97 ^a^
NCWs-H	40,834	4416 ^b^	4407.22 ^b^	6.91^b^	0.004 ^bc^	6031.85 ^bc^	0.96 ^c^
NCWs-M	40,834	4466 ^b^	4458.33 ^b^	6.94^b^	0.004 ^bc^	6168.03 ^bc^	0.96 ^c^
NCWs-L	40,834	4511 ^b^	4525.11 ^b^	6.94^b^	0.004 ^bc^	6384.19 ^b^	0.96 ^c^

Note: The different letters indicate significant differences (*p* < 0.05); the absence of letters indicates no significant differences.

## Data Availability

The original contributions presented in this study are included in the article/Supplementary Material. Further inquiries can be directed to the corresponding author.

## Supplementary Materials

The following supporting information can be downloaded at: https://www.mdpi.com/article/10.3390/plants15121851/s1, Figure S1: Variations of (A) dissolved oxygen (DO) and (B) pH in mangrove wetlands under different nitrogen input levels. Figure S2: Purification efficiency of carbon and nitrogen in layered water quality from mangrove wetlands under different nitrogen inputs. (A) NH_4_^+^-N removal rate; (B) NO_2_^−^-N concentration; (C) NO_3_^−^-N removal rate; (D) TOC removal rate. Figure S3: Pearson correlation analysis between root exudates (major categories) and N_2_O net emission, water purification efficiency, and soil nitrogen storage in mangrove wetlands (* *p* < 0.05, ** *p* < 0.01). Figure S4: Pearson correlation analysis between root exudates (subcategories) and N_2_O net emission, water purification efficiency, and soil nitrogen storage in mangrove wetlands (* *p* < 0.05, ** *p* < 0.01). Figure S5: Pearson correlation analysis between root exudates (major categories) and nitrogen metabolic functional genes in mangrove wetlands (* *p* < 0.05, ** *p* < 0.01). Figure S6: Pearson correlation analysis between root exudates (major categories) and nitrogen-metabolizing microorganisms at phylum level in mangrove wetlands (* *p* < 0.05, ** *p* < 0.01). Figure S7: Pearson correlation analysis between root exudates (subcategories) and nitrogen-metabolizing microorganisms at phylum level in mangrove wetlands (* *p* < 0.05, ** *p* < 0.01). Figure S8: Pearson correlation analysis between root exudates (major categories) and nitrogen-metabolizing microorganisms at genus level in mangrove wetlands (* *p* < 0.05, ** *p* < 0.01). Figure S9: Pearson correlation analysis between root exudates (subcategories) and nitrogen-metabolizing microorganisms at genus level in mangrove wetlands (* *p* < 0.05, ** *p* < 0.01).
