# Supplementary material for: Nitrogen Input Alters Root Exudation of Kandelia obovata and Nitrogen Cycling in Constructed Mangrove Wetlands
Source: Plants (Basel). 2026 Jun 15;15(12):1851. doi: 10.3390/plants15121851 (PMC13307435; doi:10.3390/plants15121851)
Supplement: Supplementary file 1 [file plants-15-01851-s001.zip › plants-4329037-supplementary.pdf]

Supplementary information

FIGURE

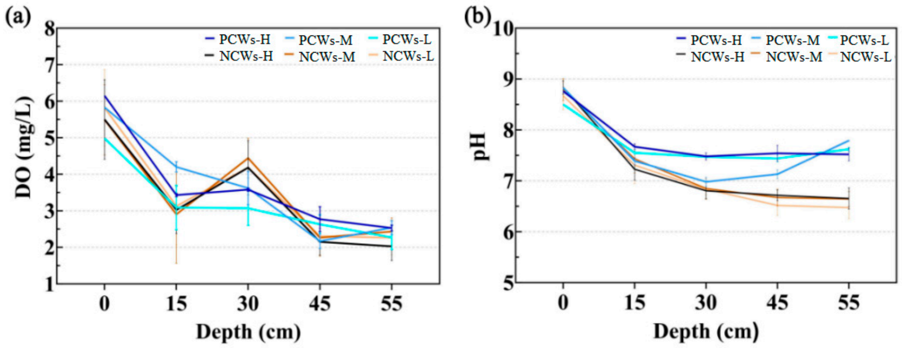

Fig. S1 Variations of (a) dissolved oxygen (DO) and (b) pH in mangrove wetlands under different nitrogen input levels.

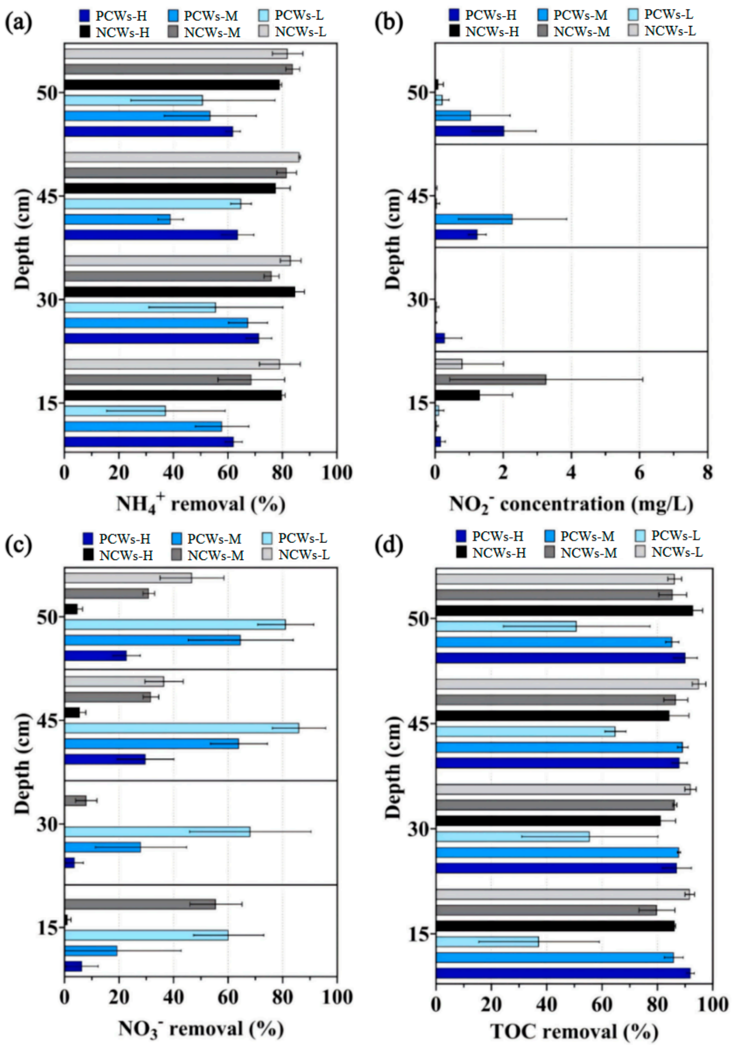

Fig. S2 Purification efficiency of carbon and nitrogen in layered water quality from mangrove wetlands under different nitrogen inputs.(a) NH_4^+-N removal rate; (b) NO_2^--N concentration; (c) NO_3^--N removal rate; (d) TOC removal rate.

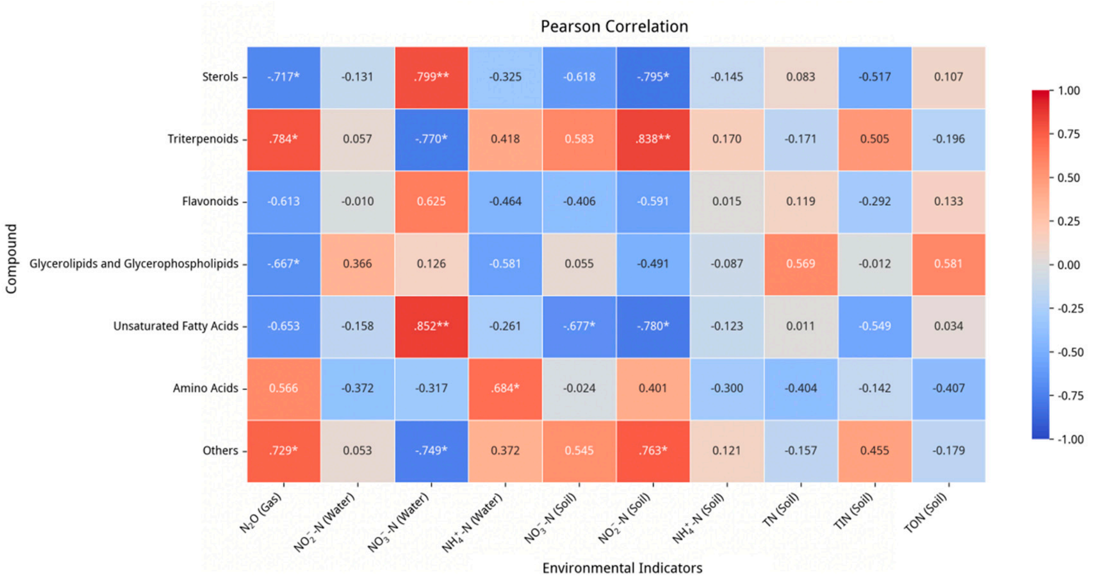

Fig. S3 Pearson correlation analysis between root exudates (major categories) and N_2O net emission, water purification efficiency, and soil nitrogen storage in mangrove wetlands (* $p < 0.05$, ** $p < 0.01$).

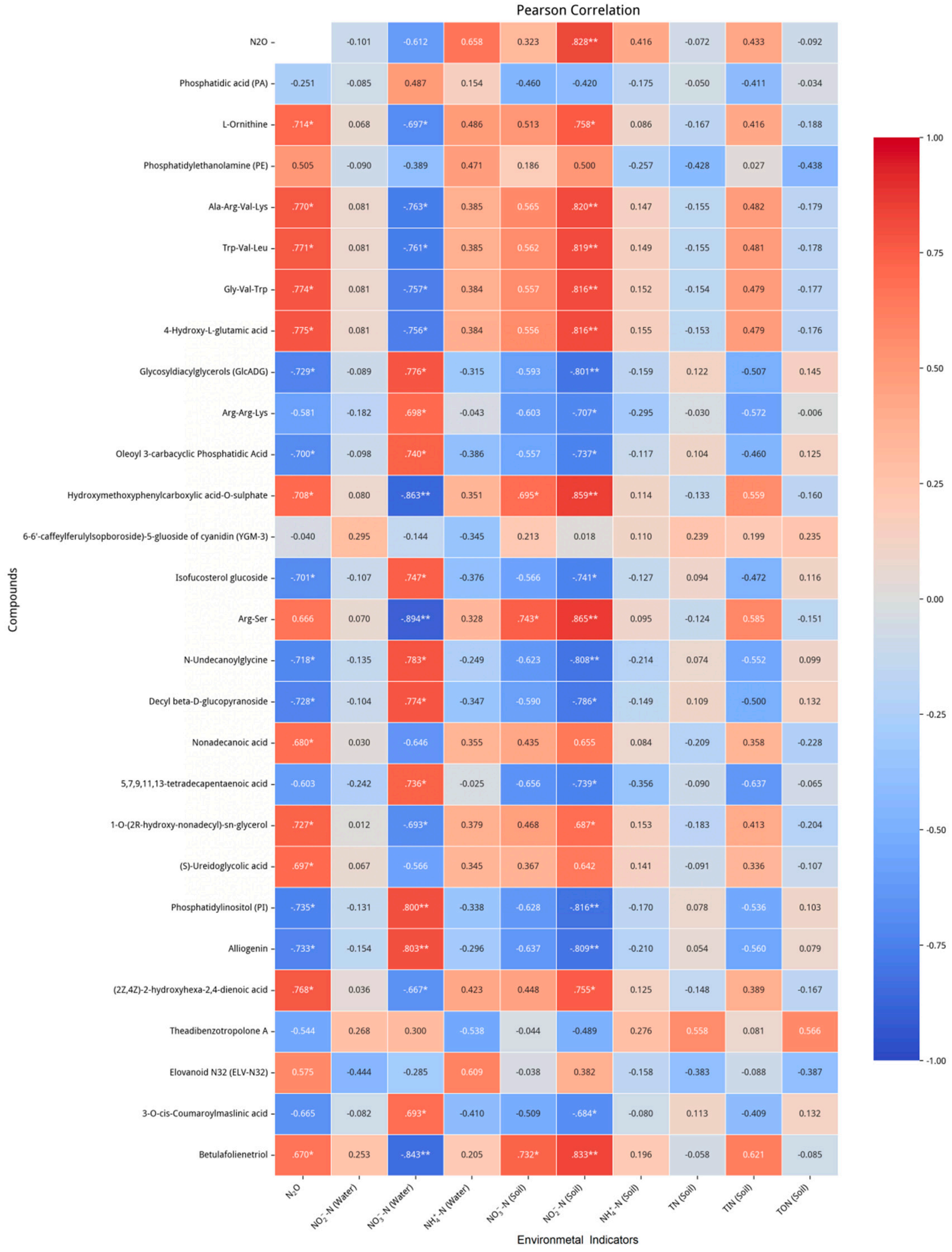

Fig. S4 Pearson correlation analysis between root exudates (subcategories) and N_2O net emission, water purification efficiency, and soil nitrogen storage in mangrove wetlands (* $p < 0.05$, ** $p < 0.01$).

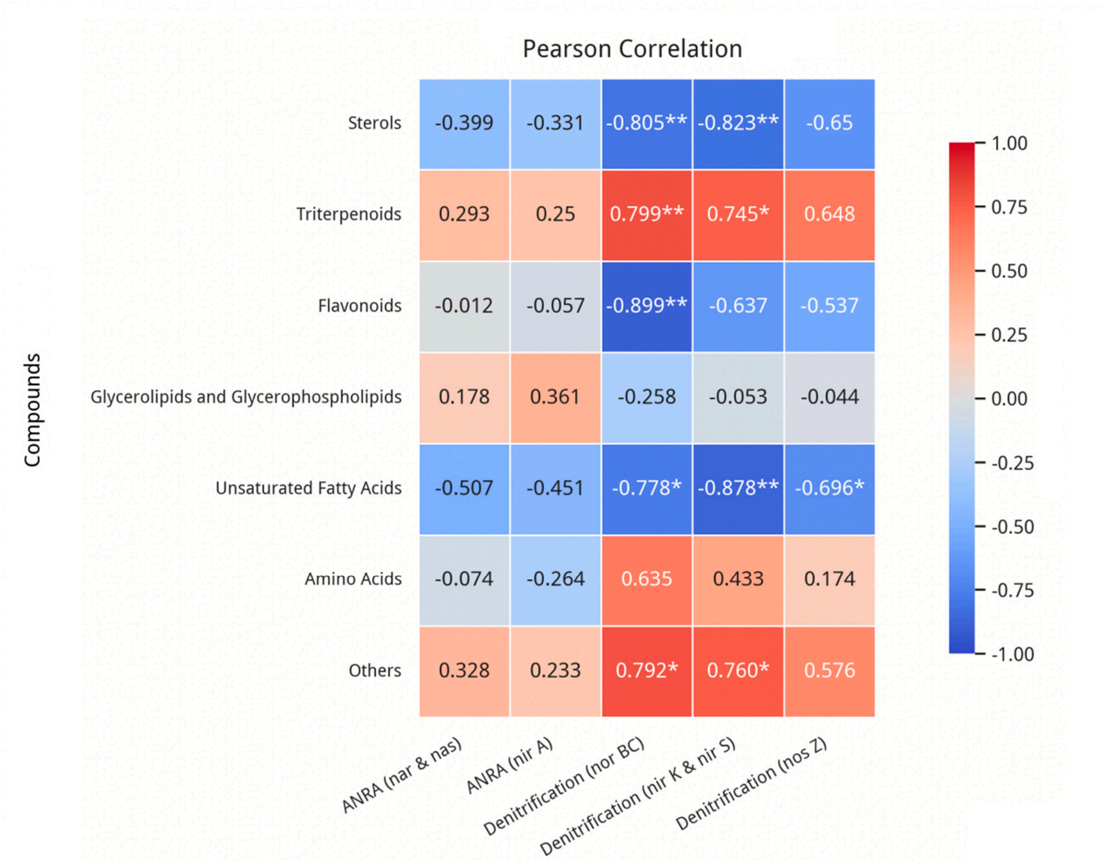

Fig. S5 Pearson correlation analysis between root exudates (major categories) and nitrogen metabolic functional genes in mangrove wetlands (* $p < 0.05$, ** $p < 0.01$).

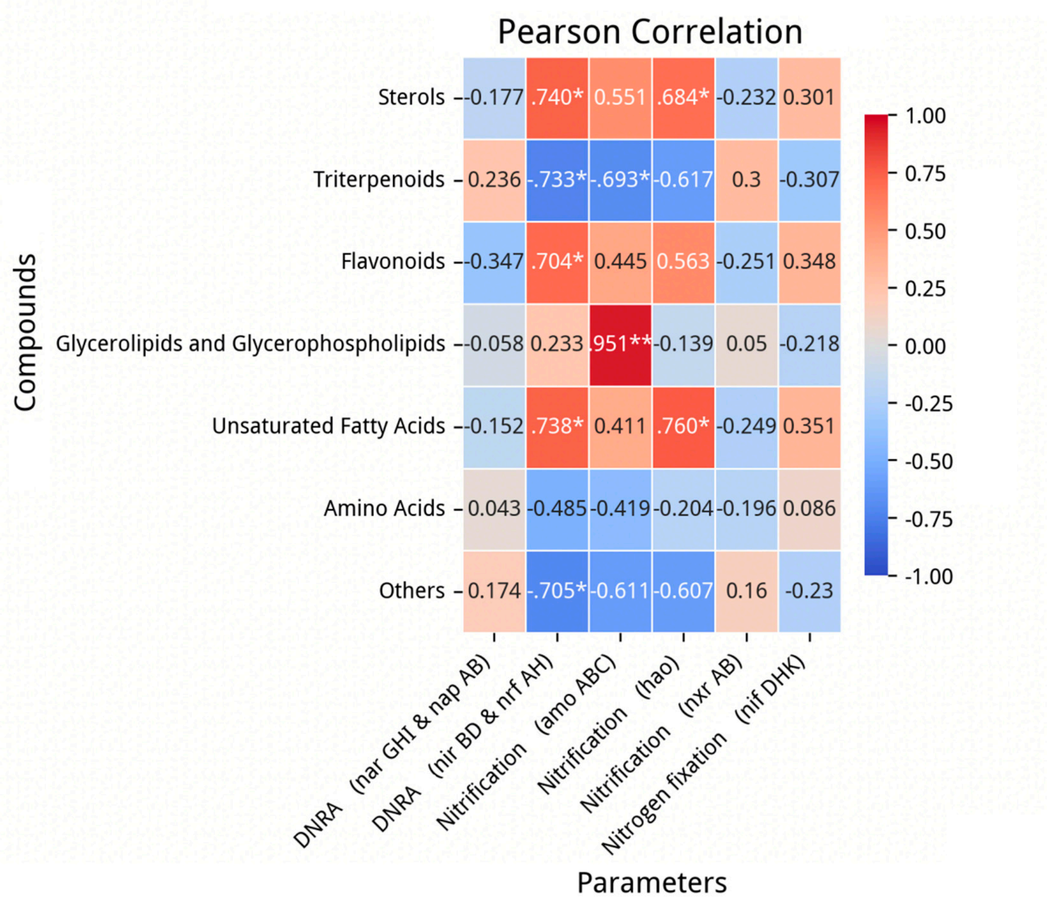

(Continued) **Fig. S5** Pearson correlation analysis between root exudates (major categories) and nitrogen metabolic functional genes in mangrove wetlands (* $p < 0.05$, ** $p < 0.01$).

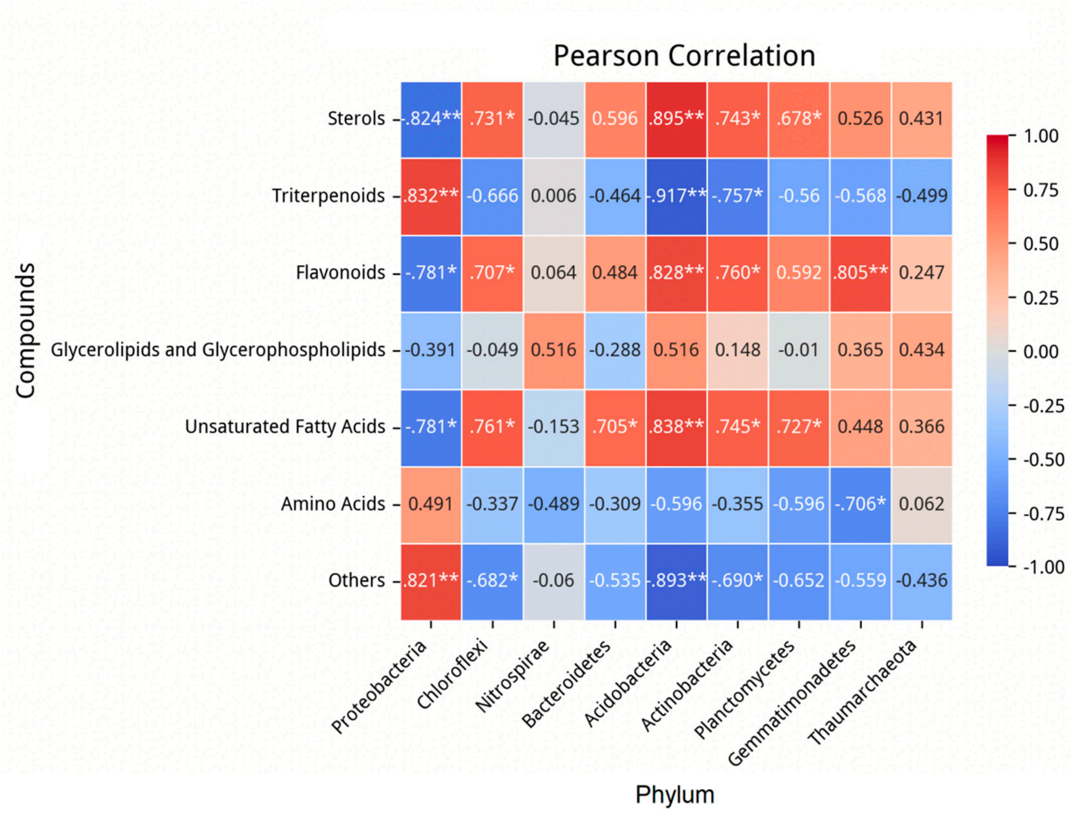

Fig. S6 Pearson correlation analysis between root exudates (major categories) and nitrogen-metabolizing microorganisms at phylum level in mangrove wetlands (* $p < 0.05$, ** $p < 0.01$).

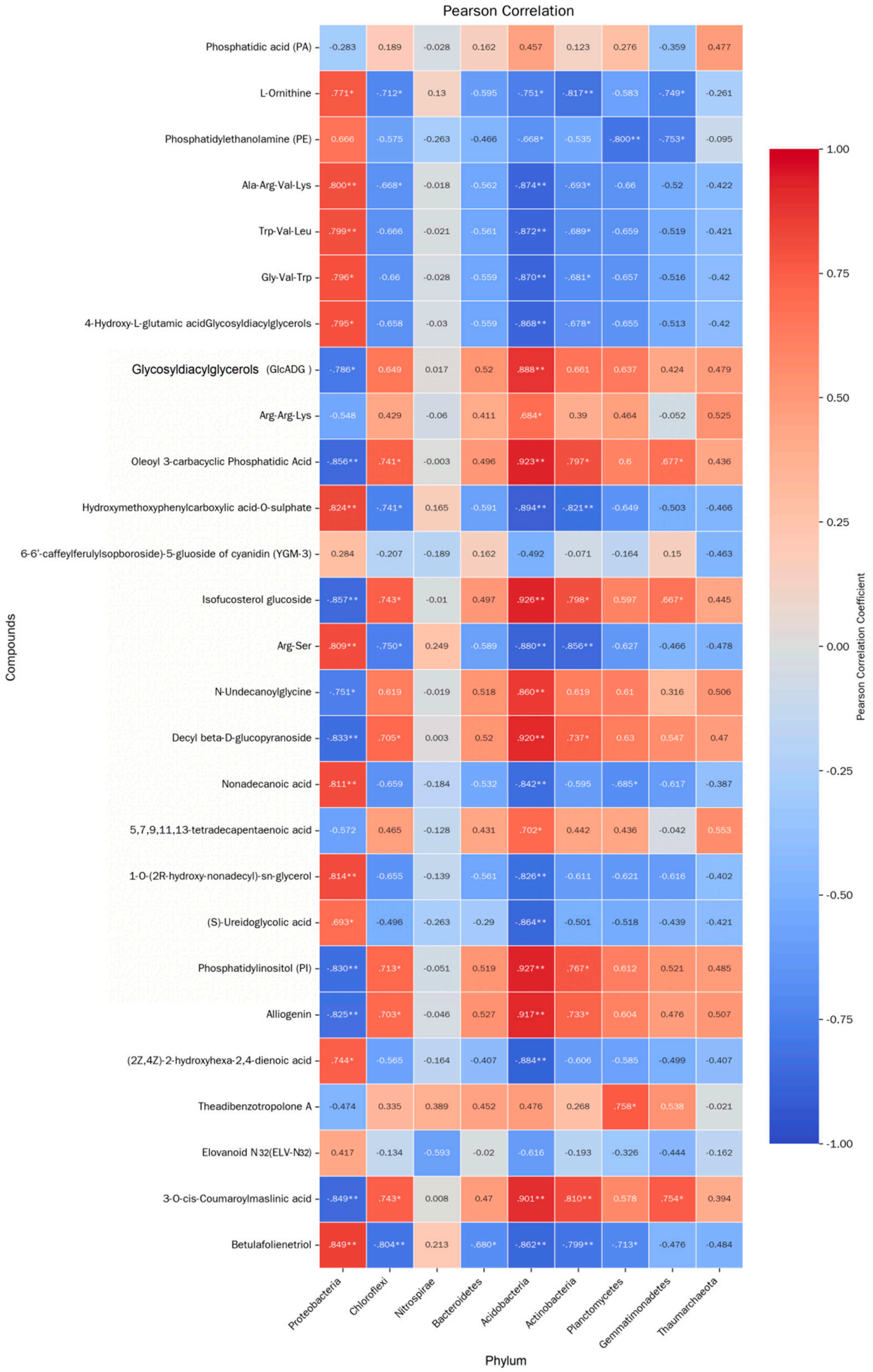

Fig. S7 Pearson correlation analysis between root exudates (subcategories) and nitrogen-metabolizing microorganisms at phylum level in mangrove wetlands (* $p < 0.05$, ** $p < 0.01$).

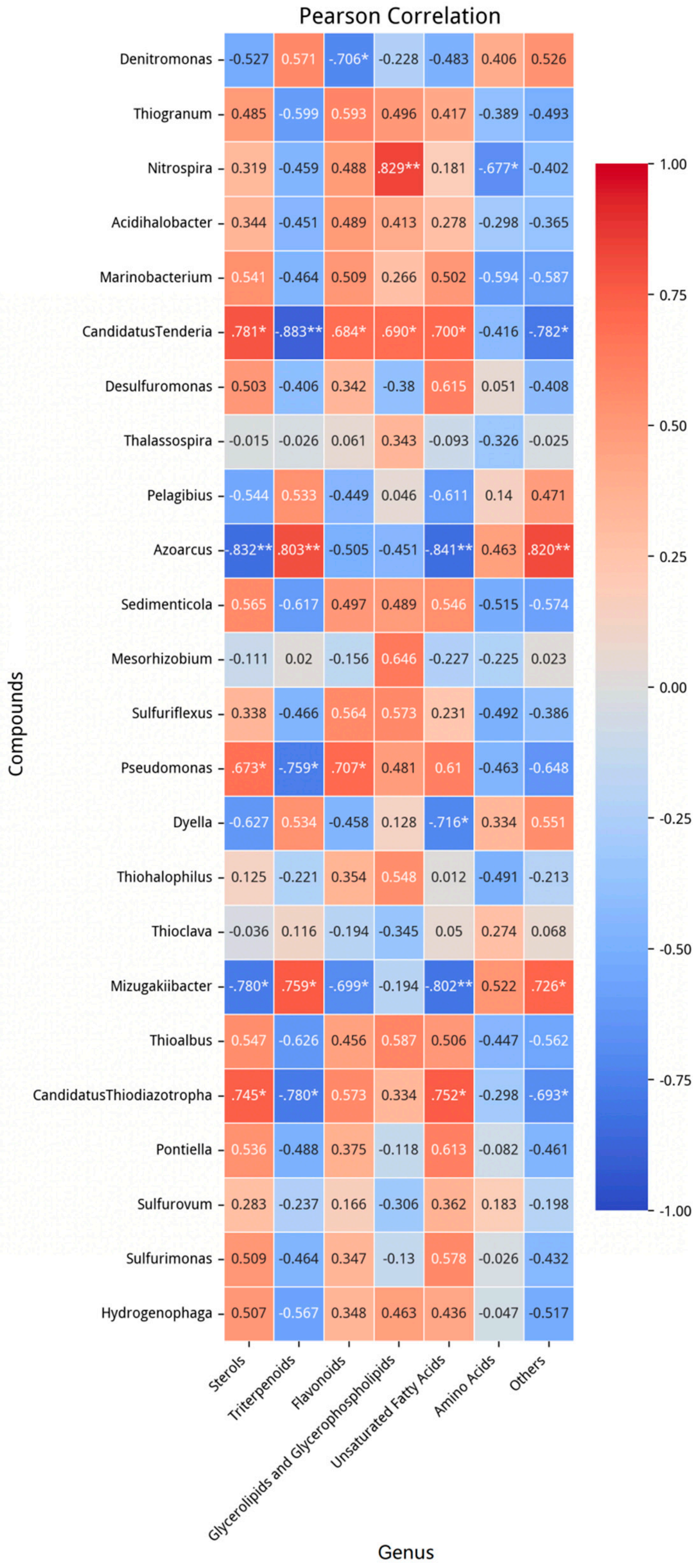

Fig. S8 Pearson correlation analysis between root exudates (major categories) and nitrogen-metabolizing microorganisms at genus level in mangrove wetlands (* $p < 0.05$, ** $p < 0.01$).

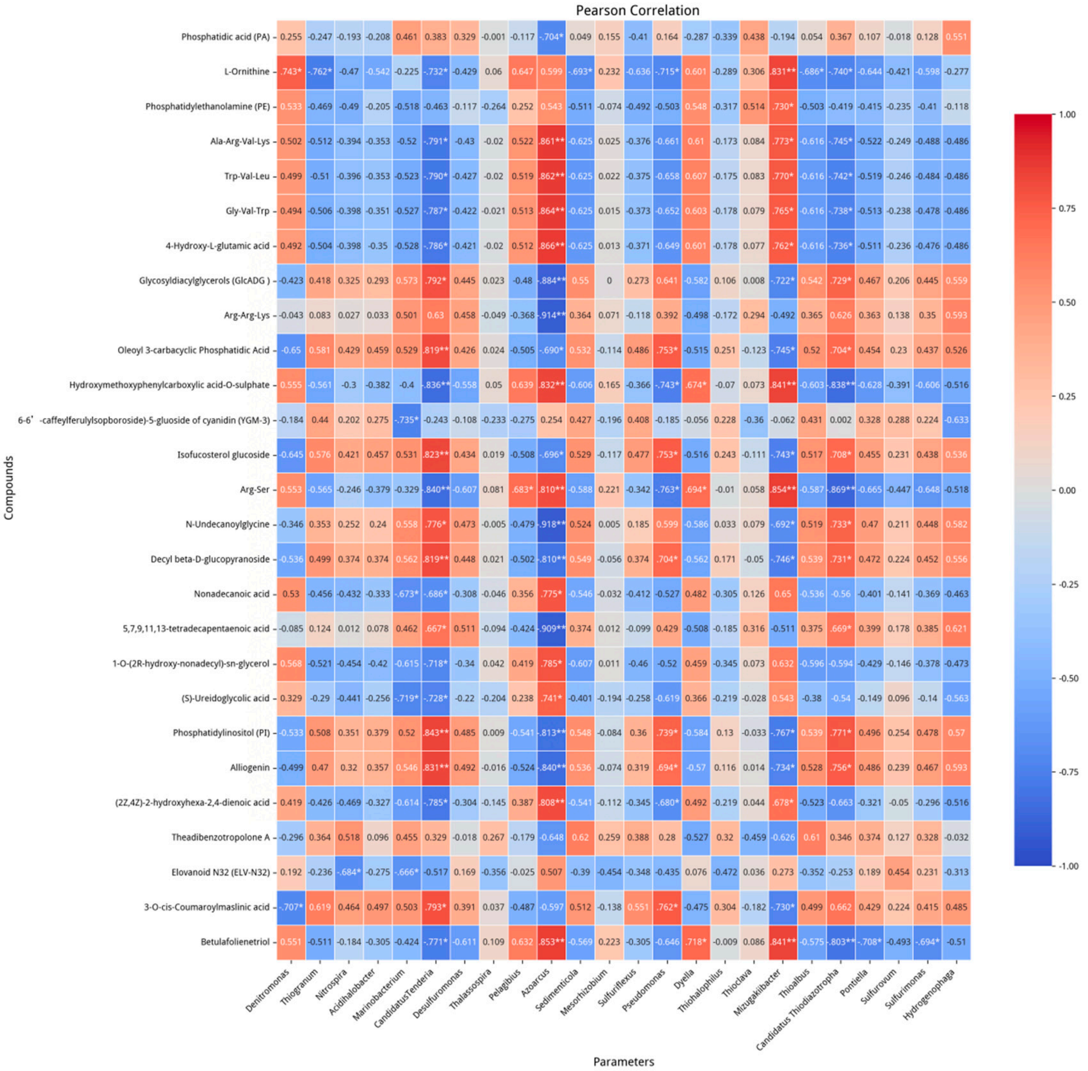

Fig. S9 Pearson correlation analysis between root exudates (subcategories) and nitrogen-metabolizing microorganisms at genus level in mangrove wetlands (* $p < 0.05$, ** $p < 0.01$).
